# Editorial: Eukaryotic Microbes Store Nitrate for “Breathing” in Anoxia

**DOI:** 10.3389/fmicb.2017.02439

**Published:** 2017-12-07

**Authors:** Anja Kamp, Peter Stief

**Affiliations:** ^1^AIAS, Aarhus Institute of Advanced Studies, Aarhus University, Aarhus, Denmark; ^2^Nordcee, Department of Biology, University of Southern Denmark, Odense, Denmark

**Keywords:** nitrogen-cycle, diatoms, foraminifers, gromiids, endobionts, marine snow, intracellular nitrate, low-oxygen environments

The microbial nitrogen cycle is one of the most complex and environmentally important element cycles on Earth, but most studies focus exclusively on the direct involvement of prokaryotes. Therefore, this Research Topic was launched to attract more attention to the role of eukaryotic microbes in the nitrogen cycle. In seven contributions, new findings on intracellular storage and the dissimilatory and assimilatory use of nitrate by eukaryotic microbes are summarized and discussed.

The Research Topic was inaugurated with a Review Article that provides an overview of the ecophysiology of nitrate storage and dissimilatory nitrate reduction by diverse marine eukaryotes (Kamp et al.). The review emphasizes that eukaryotic nitrate storage and dissimilatory nitrate reduction are far more widespread than previously envisioned. Even though this field of research is still in its infancies, recent advances further strengthen the ecological significance of this metabolic trait. Dissimilatory nitrate reduction has now been confirmed in various species, which are so far covering four out of eight major lineages in the eukaryotic tree of life, i.e., foraminifers, diatoms, fungi, and ciliates. Nitrate storage has been found in even six out of the eight lineages, i.e., foraminifers, gromiids, diatoms, fungi, dinoflagellates, haptophytes, and chlorophytes. A compilation of intracellular nitrate inventories in various sediments indicates that the eukaryotic intracellular nitrate pools vastly exceed porewater nitrate pools. Quantitative estimates on the phylogenetic partitioning of dissimilatory nitrate reduction suggest that eukaryotes may even rival prokaryotes in certain sediments.

The Review Article was followed by six Original Research Articles. Two of them focus on nitrate storage and nitrogen turnover in sinking diatom-bacteria aggregates (Kamp et al.; Stief et al.). Depending on their size and respiration activity, diatom-bacteria aggregates can develop an anoxic center even at relatively high oxygen concentrations in the surrounding water. Aggregates sinking through oxygenated waters might thus be important hot spots for anaerobic nitrogen conversions, including denitrification and thus fixed-nitrogen loss. Anoxia inside the aggregates enables nitrate-storing and aggregate-forming diatoms, such as *Skeletonema marinoi*, to perform Dissimilatory Nitrate Reduction to Ammonium (DNRA). Interestingly though, the nitrate initially stored by diatoms also becomes available to the bacterial community of the aggregate during its descent. This partially uncouples the denitrification activity by aggregate-associated bacteria from ambient nitrogen supplies. It was also speculated that intracellular nitrate not converted before the aggregates have settled onto the seafloor might fuel benthic nitrogen transformations (Marzocchi et al., 2017).

Two articles of this Research Topic deal with foraminifers, which were the first marine eukaryotes, found to perform denitrification on intracellular nitrate in anoxic conditions (Risgaard-Petersen et al., 2006). This groundbreaking finding has since stimulated research on denitrification by foraminifera and their endobionts (e.g., Piña-Ochoa et al., 2010; Bernhard et al., 2012). Here, additional research activities centering on foraminifera exposed to anoxic conditions are presented. Nitrate incorporation and subsequent assimilation by foraminifera, or possibly by their endobionts, in dysoxic or anoxic conditions has been studied by Nomaki et al. An important conclusion was that foraminiferal (intracellular) nitrate is not solely used for denitrification, but can also fuel nitrogen assimilation, even in oxygen-depleted environments. Along these lines, the *in situ* experiments with ^13^C- and ^15^N-labeled phytodetritus by Enge et al. provide insights into organic C and N uptake by calcareous foraminifera that were studied along a depth transect in sediments of the Oxygen Minimum Zone of the Arabian Sea. The study revealed that the cellular C/N uptake-ratio is lower, if foraminifers are exposed to lower oxygen concentrations. The authors discuss that the low C/N uptake-ratio is due to a greater demand or storage of organic (i.e., food-based) nitrogen in anoxic conditions and that it is potentially used (as nitrate) for denitrification and thus for energy conservation.

Gromiids are rather large, nitrate-storing protists that, similar to diatom-bacteria aggregates, may turn anoxic internally due to their own respiration activity. Sources and sinks of intracellular nitrate in gromiids and the potential role of their bacterial endobionts in the ensuing nitrate dynamics were studied by Høgslund et al. Tracer experiments with ^15^N revealed that intracellular nitrate is both taken up from the environment and produced internally, probably by bacterial endobionts performing nitrification. Anoxia triggers denitrification activity that is apparently mediated by bacterial endobionts since it can be inhibited by antibiotics.

The article by Garcia-Robledo et al. addresses the spatial-temporal dynamics of inorganic nutrients in the sediment of an intertidal mudflat with previously unseen comprehensiveness. The magnitude and relative importance of porewater, intracellular, and exchangeable pools of nitrate, ammonium, and phosphate were correlated with the seasonal dynamics in abundance and photosynthetic activity of benthic microalgae. Interestingly, exchangeable nitrate, i.e., nitrate ionically adsorbed to organic matter or clay particles, accounted for the largest fraction of the total sedimentary nitrate pool, a phenomenon so far only described for ammonium and phosphate. These results suggest that anoxic sediment layers may actually harbor two sources of nitrate, i.e., intracellular and exchangeable nitrate, which might supply electron acceptors to the microbial community.

In summary, this Research Topic reports novel findings on nitrate storage and use across a broad phylogeny reaching from diatoms (and other microalgae) to foraminifers and gromiids. The included studies address the ecophysiology of the organisms and their environmental impact on pelagic and benthic habitats as well as in anoxic micro-niches, such as diatom-bacteria aggregates or even inside of gromiids. Further, the metabolic fate of assimilated nitrogen and the biogeochemical role of intracellular vs. adsorbed nitrogen compounds in sediments were revealed. The new perspectives provided here are expected to stimulate more research on the eukaryotic players in the nitrogen cycle, especially since their ecological significance is becoming increasingly clear.

## Author contributions

All authors listed have made a substantial, direct and intellectual contribution to the work, and approved it for publication.

### Conflict of interest statement

The authors declare that the research was conducted in the absence of any commercial or financial relationships that could be construed as a potential conflict of interest.
